# Recombinant *L. lactis* vaccine LL-plSAM-WAE targeting four virulence factors provides mucosal immunity against *H. pylori* infection

**DOI:** 10.1186/s12934-024-02321-4

**Published:** 2024-02-24

**Authors:** Furui Zhang, Linhan Ni, Zhen Zhang, Xuegang Luo, Xuequan Wang, Wenmiao Zhou, Jiale Chen, Jing Liu, Yuliang Qu, Kunmei Liu, Le Guo

**Affiliations:** 1 College of Pharmacy, Ningxia Medical University, Yinchuan, 750004 China; 2 College of First Clinical Medicine, Ningxia Medical University, Yinchuan, 750004 China; 3 College of Laboratory Medicine , Ningxia Medical University, Yinchuan, 750004 China; 4 Department of Geriatrics and Special Needs Medicine, General Hospital of Ningxia Medical University, Yinchuan, 750004 China; 5 Ningxia Key Laboratory of Cerebrocranial Diseases, Ningxia Medical University, Yinchuan, 750004 China; 6 Key Laboratory of Radiation Oncology of Taizhou, Taizhou Hospital of Zhejiang Province affiliated to Wenzhou Medical University, Taizhou, 317000 China; 7 Key Laboratory of Industrial Fermentation Microbiology of the Ministry of Education, College of Biotechnology, Tianjin University of Science and Technology, Tianjin, 300457 China; 8 Ningxia Key Laboratory of Clinical and Pathogenic Microbiology, General Hospital of Ningxia Medical University, Yinchuan, 750004 China

**Keywords:** *Helicobacter pylori*, *Lactic acid bacteria*, M cell-targeting, Vaccine delivery system, Mucosal immune response

## Abstract

**Background:**

*Helicobacter pylori* (*H. pylori*) causes chronic gastric disease. An efficient oral vaccine would be mucosa-targeted and offer defense against colonization of invasive infection in the digestive system. Proteolytic enzymes and acidic environment in the gastrointestinal tract (GT) can, however, reduce the effectiveness of oral vaccinations. For the creation of an edible vaccine, *L. lactis* has been proposed as a means of delivering vaccine antigens.

**Results:**

We developed a plSAM (pNZ8148-SAM) that expresses a multiepitope vaccine antigen SAM-WAE containing Urease, HpaA, HSP60, and NAP extracellularly (named LL-plSAM-WAE) to increase the efficacy of oral vaccinations. We then investigated the immunogenicity of LL-plSAM-WAE in Balb/c mice. Mice that received LL-plSAM-WAE or SAM-WAE with adjuvant showed increased levels of antibodies against *H. pylori*, including IgG and sIgA, and resulted in significant reductions in *H. pylori* colonization. Furthermore, we show that SAM-WAE and LL-plSAM-WAE improved the capacity to target the vaccine to M cells.

**Conclusions:**

These findings suggest that recombinant *L. lactis* could be a promising oral mucosa vaccination for preventing *H. pylori* infection.

**Supplementary Information:**

The online version contains supplementary material available at 10.1186/s12934-024-02321-4.

## Background

Chronic gastritis and peptic ulcers are caused by the microaerophilic, gram-negative bacterium *H. pylori*, which invades the human stomach and duodenal mucosa [1]. The primary treatment for *H. pylori* infection involves the use of a proton pump inhibitor or ranitidine bismuth citrate in combination with clarithromycin, amoxicillin, or metronidazole [2]. The development of effective *H. pylori* vaccines is of utmost importance due to the diminishing efficacy of antibiotics due to increasing antimicrobial resistance [3]. However, there are no approved few commercial *H. pylori* vaccines currently available to our knowledge. As intriguing candidate antigens for *H. pylori* vaccines, Urease [4, 5], NAP [6], HpaA [7] and HSP60 [8, 9] proteins have recently been suggested. We have previously reported the development of a multivalent vaccine against *H. pylori* based on these virulence factors that offered therapeutic protection in Mongolian gerbils [10]. However, to make it an effective vaccine for oral delivery, and provide more robust defense against *H. pylori* infection, it is essential to design a rational mucosal vaccine delivery system.

Approximately 90% pathogenic infections, including *H. pylori*, spread via mucosal surfaces [11, 12]. Due to this, mucosal vaccination can assist in overcoming the drawbacks of the presently available injection-based vaccinations by establishing a protective immunity against these illnesses. Oral vaccinations provide significant benefits over conventional injection-only vaccines, including good safety, compliance, and ease of manufacture [13, 14]. However, there are currently few commercially available mucosal vaccines. The difficulty in delivering antigens into the mucosa, the hostile environment, obstacles in the gastrointestinal tract, and immunity of the buccal mucosa toleration have been blamed for this [15, 16].

*Lactic acid bacteria* (LAB) offer advantages as a novel oral vaccination delivery vehicle [17, 18]. LAB have been utilized for oral vaccinations against viruses and pathogens because they have great stability, are resistant to stomach acid, and is generally recognized as safe (GRAS) [19]. *L. lactis* can operate as mucosal immune adjuvants, boost the immunological potency of mucosal vaccines [20]. Additionally, the administration of modified *L. lactis* into the mucosa may effectively trigger immune responses at the mucosal and systemic levels [21]. Moreover, NICE—Nisin-Controlled gene Expression has been developed for use in *L. lactis* for the expression of foreign proteins [22].

The most important characteristic of an effective oral vaccination is to ensure that antigens are ingested and then delivered into MALT over the mucosal defense. Therefore, to construct efficient oral mucosal vaccines, M cells are appropriate targets for delivering antigens and generating a mucosal immune response [23]. M cells, which are found in the nasopharynx-associated lymphoid tissue (NALT) or the follicle-associated epithelium (FAE) of Peyer’s patches (PPs), are essential for the absorption of luminal antigens and the generation of antigen-specific immune responses in both systemic and mucosal compartments [24, 25]. In fact, M cell targeting has been attempted utilizing a variety of M cell-targeting ligands, including Co1 [26, 27], Cpe [28, 29] and CKS9 [30]. These ligands facilitate the absorption of oral vaccinations by M cells and augment the immune response specific to antigens on both systemic and mucosal surfaces.

In our earlier research, we developed a multi epitope vaccine containing four *H. pylori* virulence factors—Urease, NAP, HSP60, and HpaA, this was designated CWAE, oral CWAE with polysaccharide adjuvant (PA)-immunized mice demonstrated excellent protection against *H. pylori* infection [10]. . Unlike previous studies [31], this study uses a similar *L. lactis* surface expression system but with different combination of *H. pylori* antigens (Urease, NAP, HSP60, and HpaA) [10]. In this investigation, plSAM (pNZ8148-SAM) was used to make it easier to administer the CWAE vaccination and elicit immunological responses in the gastrointestinal tract. In addition, we engineered an LL-plSAM-WAE (pNZ8148-SAM-WAE in *L. lactis* NZ9000) that expressed the CWAE multi epitope antigens via the NICE system, and targets M cells. We investigated the protective efficacy, effectiveness of LL-plSAM-WAE in a Balb/c mouse model and investigated both systemic and mucosal responses.

## Materials and methods

### Plasmid, bacterial strains and growth conditions

*L. lactis* NZ9000 and the plasmid pNZ8148 (Zoonbio Biotechnology, China) were used in this study. *Helicobacter pylori* Sydney Strain-1 (*H. pylori* SS1) was stored in our laboratory. *L. lactis* was cultivated at 30 °C in M17 broth (Qingdao Haibo Biotechnology, China) containing 0.5% glucose (w/v) (GM17) and, where needed, supplemented with chloramphenicol (5 µg/mL) for plasmid selection. *H. pylori* SS1 were cultured on brain–heart infusion (BHI) plates (Qingdao Hope Biotechnology, China) containing 5% sterile defibrillated sheep blood and bacteriostatic under microaerophilic conditions at 37 ℃ for 4 d. The bacteria were harvested and re-suspended in normal sodium, and the final concentration was adjusted to a density of 1×10^10^ colony forming units (CFUs) per milliliter before inoculation.

### Vaccine formulation

The plSAM system, designed for *L. lactis*, is a synthetic plasmid that specifically targets M cells [31]. Its main constituent is SAM, which comprises several key elements, including the custom-designed M cell-targeting peptide Mtp containing CKS-9, Cpe and Col1, and the cA binding domain. SAM was subsequently inserted into the pNZ8148 plasmid, resulting in the construction of the plSAM plasmid. Then, the WAE gene (Urease, HpaA, HSP60, and NAP) was amplified from the pET-CWAE plasmid via PCR. Subsequently, the fragment WAE was further inserted into the plSAM plasmid named plSAM-WAE. After that, the recombinant plasmid plSAM-WAE was immediately transformed into *L. lactis* NZ9000 to construct LL-plSAM-WAE.

### Expression and identification of LL-plSAM-WAE

The LL-plSAM-WAE were cultivated on GM17 solid medium overnight in advance, and then a single colony was isolated in 5 mL GM17 liquid medium for amplification. After that, 4 mL of the amplified bacterial fluid was added to 100 mL of GM17 broth containing 5 µg/mL chloramphenicol. When the OD_600_ of the broth reached 0.6–0.8, the inducer nisin (Sigma - Aldrich, USA) was added to the culture at a concentration of 1 ng/mL expressing the SAM-WAE proteins. LL-plSAM-WAE was incubated at 30 °C until the OD_600_ reached approximately 2.0. Subsequently, the cellular samples were harvested and centrifuged, washed twice with PBS, and lysed via sonication. The lysates were mixed with 6× loading buffer and boiled in a water bath for complete denaturation. The bacterial proteins were identified through SDS‒PAGE and Western blotting. Briefly, the protein samples were separated by 12% SDS‒PAGE and transferred onto a PVDF membrane (Millipore, USA). The membrane was blocked with 5% skim milk solution at room temperature for 2 h, followed by incubation with mouse anti-WAE serum (1:2500) previously prepared in our laboratory at 4 °C overnight and washing with TBST three times. Then the membrane was incubated with HRP-labeled goat anti-mouse IgG (1:5000, Proteintech, USA) at room temperature for 1 h, and washed with TBST three times. Finally, the proteins were visualized using ECL reagent (NCM Biotech, China). Furthermore, immunofluorescence analysis was performed to verify proteins produced by LL-plSAM-WAE. Mouse anti-WAE serum (1:500) and FITC-labeled goat anti-mouse IgG (1:100, Proteintech, USA) were used to stain SAM-WAE proteins. Meanwhile, ELISA was performed to test the surface display of the SAM-WAE proteins. In brief, 100 µl/well coated solution containing *H. pylori* antibody was added to the enzyme-labeled plates for overnight at 4℃, the final concentration was 2 µg/mL. The next day, 200ul/well PBST was added to soak and wash the plate for 5 times, and 2 µg SAM-WAE protein and 1 × 10^8^ CFU LL-plSAM-WAE, LL-plSAM were added, respectively. LL-plSAM was incubated for 1 h and then, anti-WAE serum (1:2500) was added and incubated for 1 h. Finally, HRP labeled anti-mouse IgG was used to detected.

### Immunization protocol and sample collection

Animal experimentation protocols were approved by the Animal Ethical and Experimental Committee of Ningxia Medical University. A total of 18 six-to-eight-week old male SPF Balb/c mice were randomly divided into 3 groups, and orally administered 1 × 10^9^ CFU/300 µL LL-plSAM-WAE, *L. lactis* NZ9000 or PBS on weeks 1, 2, 3 and 4. One week after the final vaccination, blood, spleen and MLN samples of mice were collected for subsequent testing. The experimental program was executed as described in Fig. 5a. The procedure of the protective model is shown in Figs. 6a and 7 days after the last oral vaccination, mice received 300 µL of an *H. pylori* suspension at 31, 33 and 35 days. Then, 15 days after the last *H. pylori* infection, the immunized and control mice were sacrificed for evaluation of *H. pylori* infection.

### Measurement of antigen-specific antibody in the serum

The mice were sacrificed one week after the last immunization, blood was taken through the orbital vein, allowed to stand for 30 min at room temperature and centrifuged at 3000 rpm for 15 min to collect serum. The antigens of Urease, UreA, UreB, HpaA and NAP were separated on SDS‒PAGE gels and transferred onto PVDF membranes. Then, the serum was analyzed and incubated with HRP-labeled goat anti-mouse IgG. Additionally, enzyme-linked immunosorbent assay measurements of antigen-specific antibodies were made. In brief, 96-well microplates were coated at 4 °C for an overnight period with 5 µg/well SAM-WAE, urease, NAP, HSP60, HpaA, or BSA. The plates were washed and blocked with 3% BSA in PBS. Then the plates were washed and incubated with 100 µL of mouse serum at 37 °C for 1 h. Before being measured, the serum was diluted to a concentration of 1:500 after being separated from mice that had been inoculated with either LL-plSAM-WAE or *L. lactis* NZ9000. After washing, HRP-conjugated goat anti-mouse (1:1000, Proteintech, USA) was added, and the plates were incubated again for 1 h. The color reaction based on TMB (Solarbio, China) was terminated after incubation for 15 min at room temperature by the addition of 50 µL of H_2_SO_4_ (1 M), and the absorbance at 450 nm was measured by a microplate reader (Thermo Fisher Scientific, USA).

### Immunofluorescence of the spleen

A crucial sign of specific immunity is the presence of CD4^+^ and CD8^+^ T cells. Each spleen had tissue removed, which was then embedded in paraffin after being preserved in 4% paraformaldehyde. Then frequencies of CD4^+^ and CD8^+^ cells were detected [32].

### Flow cytometry analysis on mesenteric lymph nodes and spleen tissue T cells

Vaccinated mice were sacrificed, and the mesenteric lymph nodes (MLNs) and spleens were harvested. By pulverizing the tissue through a 40 μm cell strainer, lymph nodes and spleens were produced as single-cell suspensions. ACK buffer (HyClone) was used to lyse red blood cells, which were then centrifuged and suspended in RPMI 1640 medium (Basal Media) supplemented with 10% FBS (BI, Israel), and 1% streptomycin/penicillin. Single-cell suspensions were directly stained for flow cytometric analysis. Before intracellular cytokine staining, cells were stimulated in BFA and cell stimulation cocktail (Invitrogen) for 6 h. Next, the cells were stained for extracellular markers, fixed and permeabilized with Intracellular Fixation/Permeabilization Buffer (Invitrogen). Rat anti-mouse CD3 (clone 17A2), CD4 (clone GK1.5), IFN-γ (clone XMG1.2), IL-4 (clone 11B11), and IL-17 A (clone TC11-18H10.1) antibodies were purchased from BioLegend (San Diego, CA, USA). Flow cytometry was performed on a FACS Celesta flow cytometer (BD Biosciences, USA).

### ELISPOT

Utilizing a mouse IFN-γ precoated ELISPOT kit (Mabtech AB, Sweden), ELISPOT experiments were carried out. Briefly, the plate was incubated at room temperature for 2 h with medium containing 10% serum. A total of 3 × 10^5^ lymphocytes purified from vaccinated mouse spleens were treated with 10 µg/mL CWAE (Urease, NAP, HpaA, HSP60) antigen [10] or 10 ng/mL PMA. Then, the cells were incubated in RPMI-1640 at 37 °C for 30 h. Following a wash, the plates were incubated with a detection antibody at 37 °C for two hours. After washing, the plates were incubated with streptavidin-ALP for 1 h at 37 °C. Finally, stop color development by washing extensively in tap water. Plates were counted using an ELISPOT-reader (AID).

### Analysis of M cell-targeting properties

The M cell targeting ability of LL-plSAM-WAE was investigated using closed ileum loop experiments, which were modified from the procedures outlined in other reports [33, 34]. In brief, 100 µL of LL-plSAM-WAE, 100 µg/mL SAM-WAE protein, or 100 µg/mL WAE protein was injected into the ileum loops, as appropriate. The loops were incubated, washed, fixed, and cryo-sectioned. Alexa Fluor 647 goat anti-rabbit IgG antibody and rabbit anti-WAE antibody were used to stain the sections. The Alexa Fluor 488 anti-Gp2 monoclonal antibody was used to identify M cells, and DAPI (Sigma, USA) was utilized to identify the nuclei. Finally, the cells were detected using confocal laser scanning microscopy (LSM900, Carl Zeiss AG).

### Efficiency of removal of *H. pylori* infection

Following oral vaccination, the assessment of *H. pylori* infection was conducted using quantitative PCR (qPCR) and a quick urease test. The stomach tissue was taken aseptically, divided into 3 parts along the large bend of the stomach, and the contents were removed. After weighing the first portion of stomach tissue, 0.5 mL normal saline was added and homogenized with a homogenizer for CFU assays or urease activity detection. The second portion of stomach tissue was immersed in 10% formaldehyde solution and fixed for histopathological examination. The third part of stomach tissue was immersed in 1.5 mL tube with 1 mL normal saline and temporarily stored in liquid nitrogen for qPCR detection. For CFU assays, the gastric tissues weighted and then subjected to homogenization, and by serial dilution. The samples (100 µl) was plated onto BHI blood plates (Qingdao Hope Bio-Technology, China) supplemented with antibiotics. Then, *H. pylori* DNA in stomach was extracted by bacterial genome extraction kit (TIANGEN DP302, China) and quantified by real-time PCR method. The primers are as follows: *SSA* gene forward, TGGCGTGTCTATTGACAGCGAGC, reverse, CCTGCTGGGCATACTTCACCATG. *GAPGH* gene forward, GGGGGTAGGAACACGGAA, reverse, AAGGGTGGAGCCAAAAGG. In the rapid urease test, a specimen of stomach tissue was submerged in a solution specifically designed for the test, known as the RUT solution [35]. Subsequently, the sample was subjected to incubation at 37 °C for 4 h. The measurement of absorbance was performed at a wavelength of 550 nm.

### Immunohistochemical investigation

HE staining, inflammatory scores, and immunohistochemistry studies were performed on the stomach tissue. Briefly, 10% neutral formaldehyde solution was used to fix sections of stomach tissues before they were embedded in paraffin. HE was used to stain the sections, and gastritis was assessed as previously described [36].

### ELISA was used to detect *H. pylori* antigen-specific antibodies

Serum IgG and mucosal secretory IgA (sIgA) concentrations were measured using ELISA. Before testing, the samples were briefly diluted with PBS. Microplates containing 100 µL of diluted samples and 5 µg/well of *H. pylori* lysates were coated overnight at 4 °C. HRP-conjugated goat anti-mouse IgG (31,430, Thermo Fisher) and sIgA (ab97235, Abcam) were used after the plate had been cleaned with PBST. Tetramethylbenzidine (Solarbio, China) was used to view the plates after washing for 15 min in complete darkness. Finally, a solution of 2 M sulfuric acid (Solarbio, China) was used to terminate the process. A microplate reader was used to measure the absorbance at 450 nm.

### Identification of lymphocyte reactions unique to *H. pylori*

Lymphocytes were isolated from the mouse spleen after it was removed. Following this, lymphocytes were cultivated with *H. pylori* lysates (5 µg/mL) for 72 h, after which the supernatant was collected to quantify cell proliferation using the CCK-8 test and to measure the levels of numerous cytokines (IL-4, IFN-γ, and IL-17) by ELISA.

### Statistical analysis

GraphPad Prism 8.0 software was used for statistical analysis. The results are presented as the mean ± standard deviation (SD). A t test was used to assess statistical significance. *p < 0.05; **p < 0.01, ***p < 0.001.

## Results

### Construction and verification of plSAM and plSAM-WAE plasmids

**Fig. 1:**
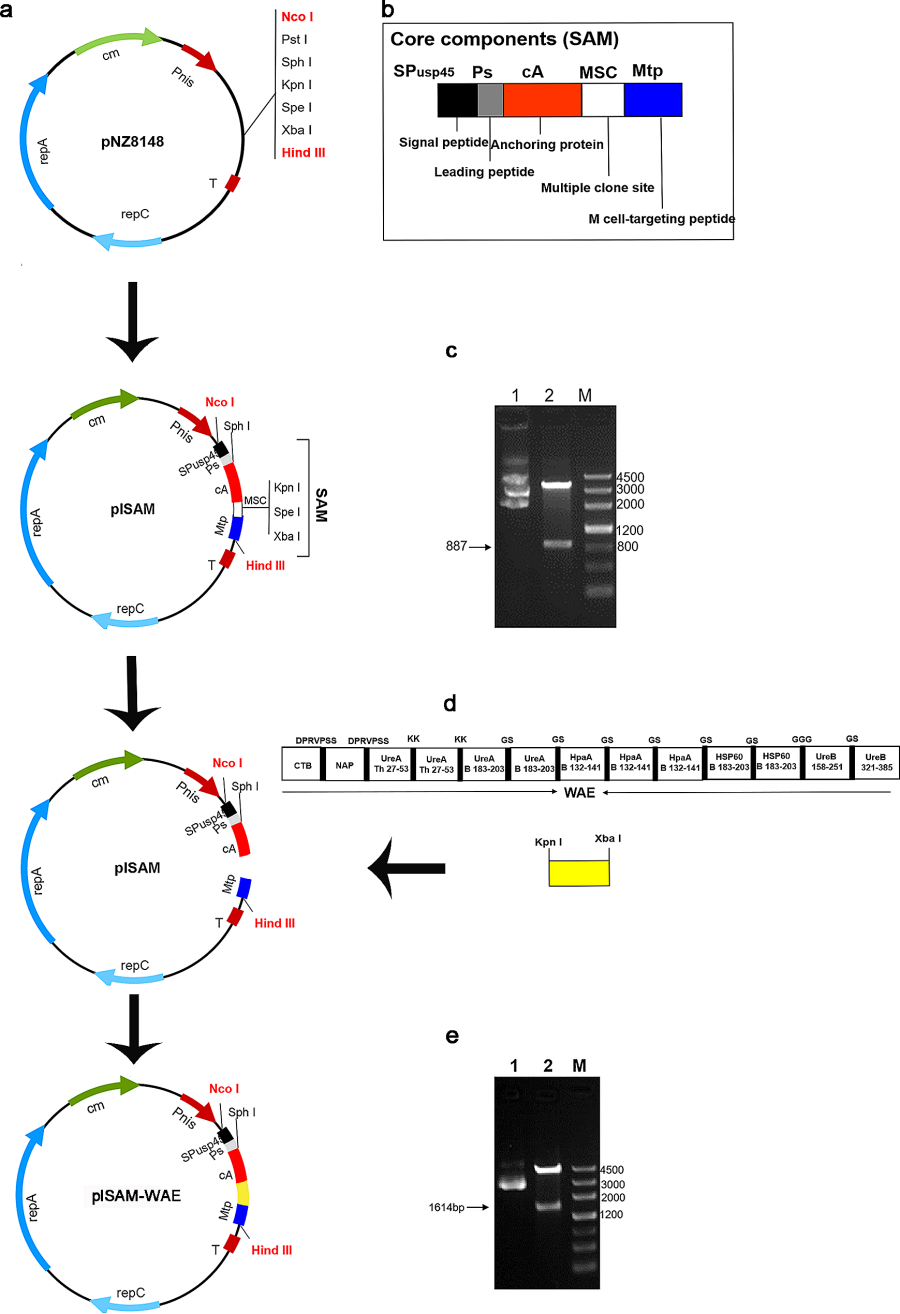
Plasmid creation and identification for plSAM-WAE. (**a**) The process for creating the plSAM-WAE plasmid. (**b**) The SAM structure. (**c**) Testing the plasmid plSAM. Plasmid plSAM is in lane (1) Nco I and Hind III digest plSAM is in lane (2) (**d**) The WAE vaccine composition. (e) plSAM-WAE’s verification is item. The plasmid plSAM-WAE is in lane 1. Plasmid plSAM-WAE digested in Lane 2

The core component of SAM (Fig. 1b), was synthesized and inserted into the plasmid pNZ8148 to create plSAM (Fig. 1a). It was digested with Nco I and Hind III, and the resultant 887 bp fragment roughly matched the SAM gene (Fig. 1c). Meanwhile, the WAE (Fig. 1d) fusion gene was amplified and introduced into plSAM as plSAM-WAE (Fig. 1a). Then the plasmid was validated by enzyme digestion and gene sequencing, and the expected fragment was observed by gel electrophoresis (Fig. 1e). These results confirmed the successful construction of plSAM and plSAM-WAE.

### Confirming the expression of *H. pylori* antigens on *L. lactis*

**Fig. 2:**
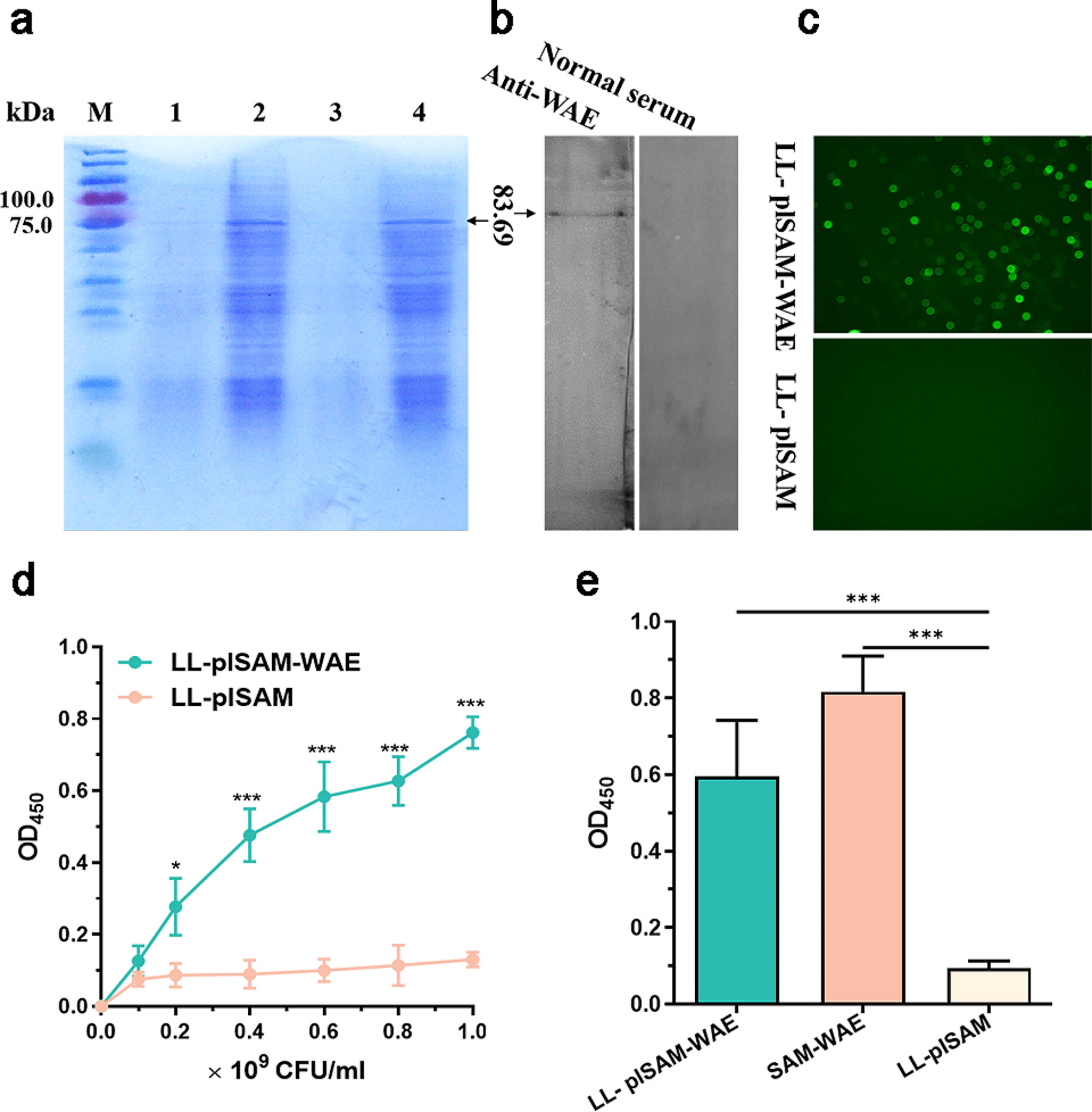
Expression and identification of LL-plSAM-WAE. (**a**) SDS‒PAGE. M, protein marker; Lanes 1 and 3 included the supernatant of LL-plSAM-WAE after induction with nisin, whereas lanes 2 and 4 contained the lysate supernatants of LL-plSAM-WAE after induction with nisin. (**b**) Western blot. The presence of the SAM-WAE protein may be identified by the use of a mouse WAE antibody. (**c**) Immunofluorescence examination. (**d**) Various doses of LL-plSAM-WAE or LL-plSAM were coated on ELISA plates. (**e**) SAM-WAE (5 µg/mL), LL-plSAM (5 × 10^8^ CFUs/well), and LL-plSAM-WAE (5 × 10^8^ CFUs/well) were coated on ELISA plates

After treatment with nisin, the results obtained from SDS‒PAGE analysis indicated that recombinant *L. lactis* successfully produced fusion proteins of SAM-WAE, with a molecular weight of 83.69 kDa (Fig. 2a). Moreover, the expression of SAM-WAE was confirmed through western blotting using mouse anti-WAE serum, as evidenced by the presence of a specific band (Fig. 2b). Conversely, negative results were observed in the lane containing normal mouse serum (Fig. 2b). Additionally, the use of specific antibodies for immunolabeling proved to be an effective method for detecting expression proteins. In this regard, green fluorescence was observed in the LL-plSAM-WAE group, while no fluorescence was detected in the LL-plSAM group (Fig. 2c). We deposited LL-plSAM-WAE, LL-plSAM, and SAM-WAE into ELISA plates at different concentrations, demonstrating that SAM-WAE was expressed by LL-plSAM-WAE (Fig. 2d-e).

### Vaccination with LL-plSAM-WAE induced T-cell immune responses

**Fig. 3:**
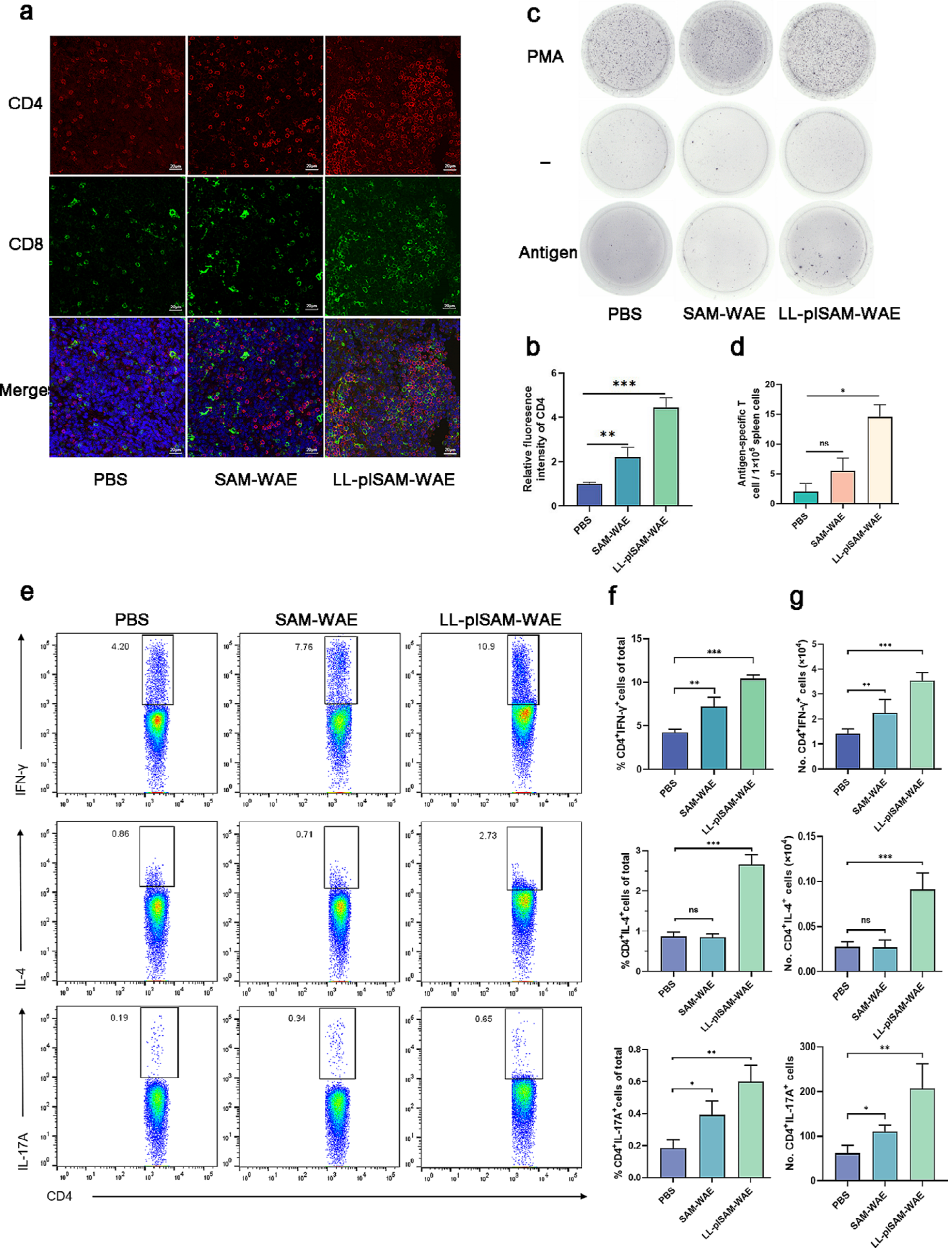
Detection of T-cell immune responses in the spleen. One week following the last immunization, spleens were taken. (**a**) The marginal zones of spleen sections from the PBS, SAM-WAE, and LL-plSAM-WAE groups were immunofluorescently stained in a representative manner. (**b**) The Relative fluoresence intensity of CD4. (**c**) The single cell suspensions from the spleen that were activated in vitro with 10 µg/mL CWAE are shown in representative ELISPOT data. (**d**) The quantity of splenic IFN-γ-producing cells that are antigen specific. (**e**) Representative FACS plots in the spleen are shown. (**f**) A total of 10^4^ CD4^+^ cells were used as a baseline to standardize the proportion and quantity of IFN-γ^+^, IL-4^+^, and IL-17^+^ cells in the spleen (**g**)

Immunofluorescence was used to determine the frequency of splenic CD4^+^ and CD8^+^ T cell responses in the PBS, SAM-WAE, and LL-plSAM-WAE groups one week after the last vaccination (Fig. 5a). As shown in Fig. 3a, the proportion of CD4^+^ and CD8^+^ T lymphocytes considerably increased compared to other groups after oral vaccination with LL-plSAM-WAE, which caused a greater frequency of CD4^+^ and CD8^+^ T cells in the marginal zone of the spleen. Meanwhile, we observed that the CD4^+^ T-cell response was relatively dominant (Fig. 3a-b). It has been suggested that the Th1/Th2/Th17 T-cell immune response promotes the best defense against *H. pylori*. Consequently, to analyze whether LL-plSAM-WAE could boost cellular immune responses, we detected the types of Th cells. The ratios of IFN-γ^+^, IL-4^+^, and IL-17 A^+^ T-cell among splenic CD4^+^ T cells increased in the LL-plSAM-WAE group compared with the PBS group (Fig. 3e-g). The results showed that Th1/Th17 immune responses were activated after oral administration of SAM-WAE (Fig. 3e-g). Moreover, IFN-γ^+^, IL-4^+^, and IL-17 A^+^ levels were considerably elevated in the mesenteric lymph nodes of mice given the LL-plSAM-WAE vaccine (Additional file 1: Figure S1), demonstrating the migration of Th1/Th2/Th17-type memory T cells. We also investigated the frequency at which spleens produced IFN-γ-producing cells specific to the CWAE antigen using ELISpot. Oral administration of free SAM-WAE generated 7 antigen-specific IFN-γ-producing cells per 500,000 spleen cells, as illustrated in Fig. 3c-d. However, in the LL-plSAM-WAE group, the frequency of antigen-specific IFN-γ-producing cells was considerably higher, demonstrating that the LAB delivery system significantly strengthened the IFN-γ-cell response.

### LL-plSAM-WAE or SAM-WAE targets M cells effectively

**Fig. 4:**
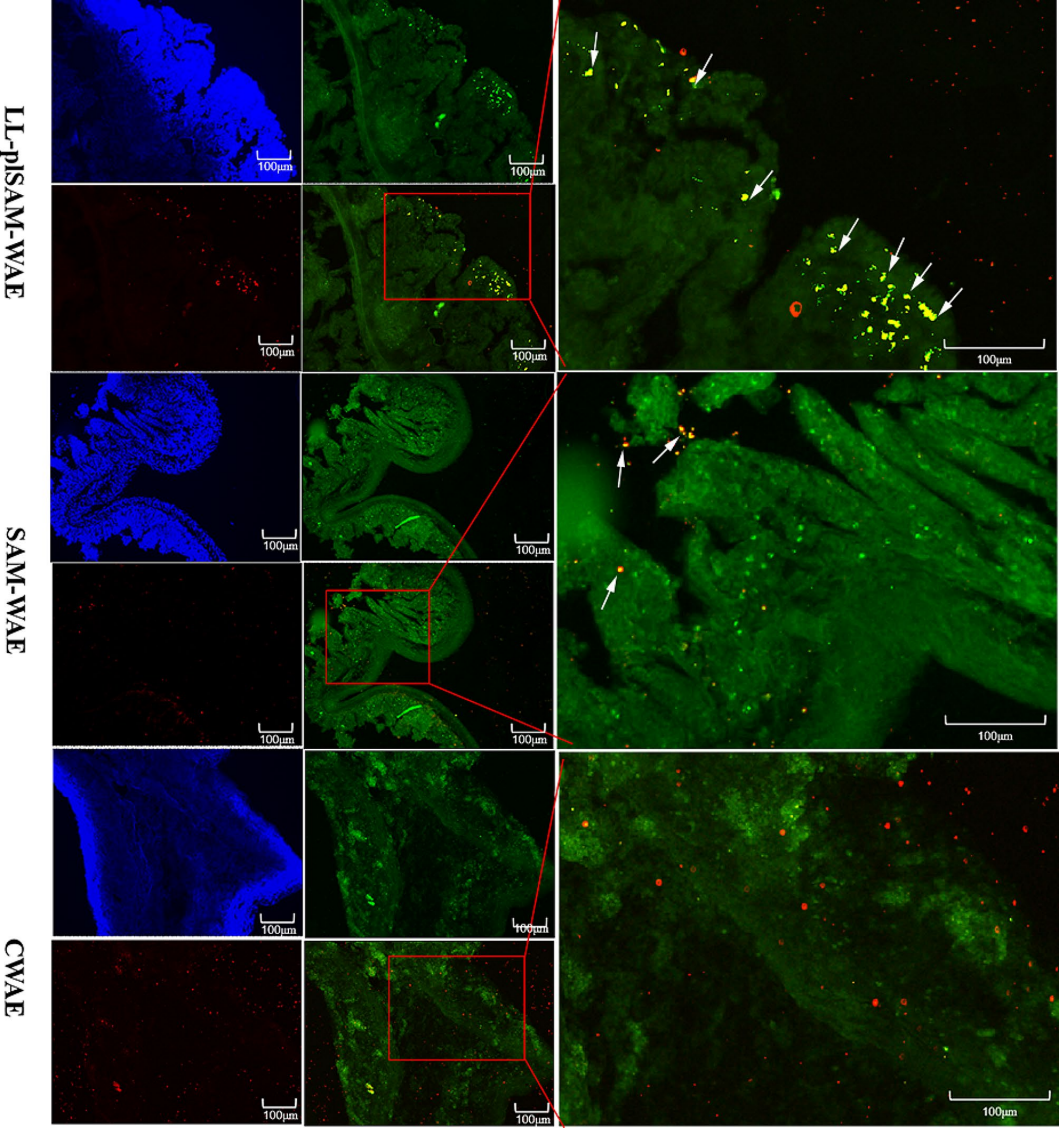
M cell-targeting detection. Red signal indicates fluorescent secondary antibody-labeled recombinant protein SAM-WAE, green signal indicates FITC-labeled M cells, and blue signal indicates DAPI labeled nuclei. White arrows indicate the co-localization signals for antigens that were directed toward M cells

We next tested whether LL-plSAM-WAE and SAM-WAE targeted M cells using the closed ileum loop model. LL-plSAM-WAE, SAM-WAE protein, or CWAE protein were each injected into naïve mice ileum loops, and the fluorescent signals were evaluated by confocal microscopy (Fig. 4). FITC-labeled anti-Gp2 was used to mark the M cells in PPs, and red light indicates fluorescent secondary antibody-labeled recombinant protein SAM-WAE. There were noticeably more yellow puncta, which indicate the co-localization of SAM-WAE and M cells, in the group treated with LL-plSAM-WAE or SAM-WAE protein (Fig. 4). In contrast, fewer puncta were observed in Peyer’s patches treated with CWAE protein (Fig. 4). These findings suggested that the LL-plSAM-WAE or SAM-WAE protein targeted M cells effectively due to the extra SAM component.

### BALB/c mice immunized with LL-plSAM-WAE produced neutralizing antibodies

**Fig. 5:**
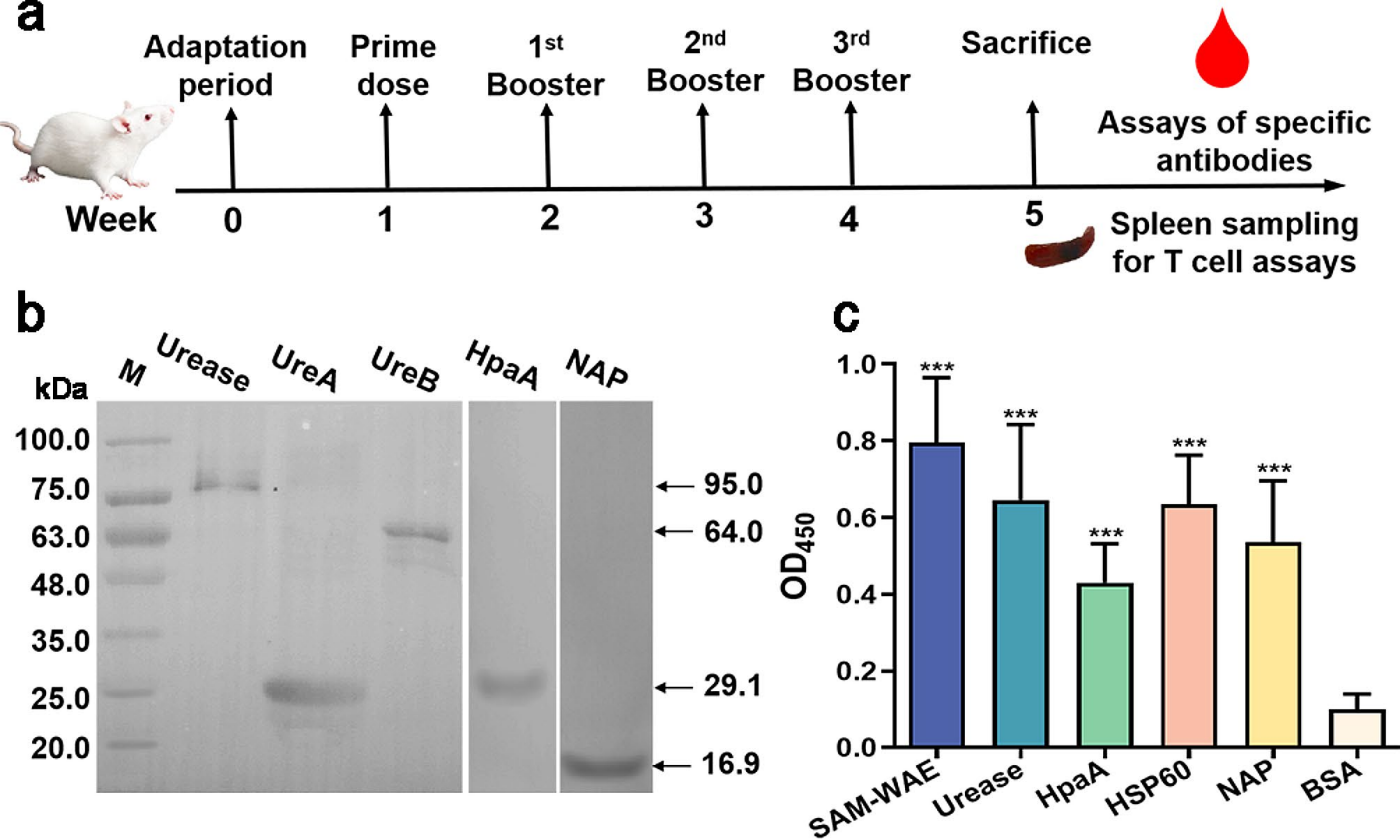
Antiserum specificity test for LL-plSAM-WAE. Antisera of mice induced by LL-plSAM-WAE via oral vaccination were collected to detect anti-*H. pylori* neutralizing antibodies. (**a**) The schedule of vaccination of BALB/c mice. (**b**) Western blot results showed that the antiserum obviously recognized the antigens Urease, UreA, UreB, HpaA and NAP. (**c**) Before ELISA, the antiserum was diluted to a concentration of 1:500, and the plates were coated with 5 µg/mL concentrations of SAM-WAE, urease, HpaA, HSP60, NAP and BSA

Effective vaccines are urgently needed to generate anti-*H. pylori* neutralizing antibodies. BALB/c mice were vaccinated with LL-plSAM-WAE, and serum was collected at one week after the last vaccination (Fig. 5a). Our results showed that the antiserum obviously recognized the antigens Urease, UreA, UreB, HpaA and NAP (Fig. 5b). And serum from mice vaccinated with LL NZ9000 as negative controls (Additional file 1: Figure S2). Meanwhile, ELISA performed with the same antiserum revealed a similar outcome (Fig. 5c). This evidence proved that antiserum induced by LL-plSAM-WAE possessed high specificity for different *H. pylori* virulence factors.

### Protective effect and histopathological analysis after oral vaccination

**Fig. 6:**
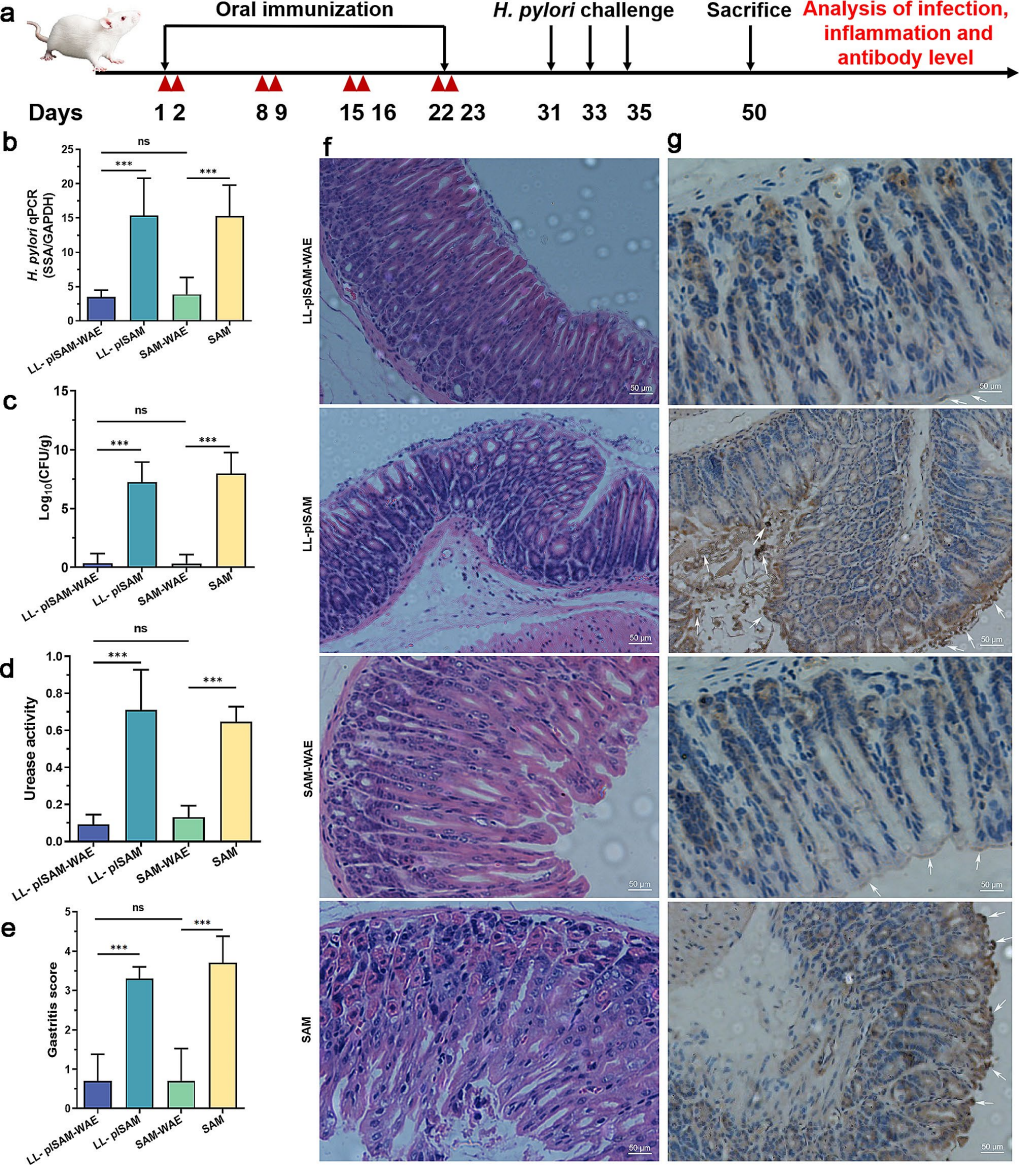
Gastric histology and protective effects were investigated. (**a**) Experimental protocol for preventing the efficacy of recombinant *L. lactis* vaccination in the treatment *H. pylori* infection in BALB/c mice. (**b**) qPCR analysis of *H. pylori*. (**c**) CFU counting of bacterial colonies per mouse stomach. (**d**) Detection of urease activity at OD_550_ nm. (**e**-**f**) Gastric histological characterization (HE staining 100× and inflammation score). (**g**) Immunohistochemistry of the gastric tissue (100×). Tissue sections were counted for positive spots, which indicate the number of *H. pylori-* positive cells

Mice were orally immunized with LL-plSAM-WAE or SAM-WAE and then infected with *H. pylori* to assess the protection of oral vaccination (Fig. 6a). Then, the stomach of each mouse was removed and used to measure the *H. pylori* burden using various techniques. According to the results of the quick urease test (Fig. 6d), *H. pylori* quantitative culture (Fig. 6c), and RT‒qPCR (Fig. 6b), oral vaccination with LL-plSAM-WAE or SAM-WAE generally reduced the *H. pylori* burden and urease activity compared to the other groups. Notably, oral vaccination resulted in dramatic post immunization stomach inflammation and an elevated level of leukocyte infiltration (Fig. 6e, f). Additionally, the inflammation between the LL-plSAM-WAE group and the SAM-WAE group did not vary significantly. Using anti-*H. pylori* antibody in IHC analysis, it was discovered that stomach tissue samples from the LL-plSAM, and SAM groups had *H. pylori* colonization. Intriguingly, only a small quantity of *H. pylori* was discovered in the LL-plSAM-WAE and SAM-WAE groups, demonstrating the effectiveness of these treatments in preventing *H. pylori* invasion (Fig. 6g).

### *L. lactis* LL-plSAM-WAE or SAM-WAE protein improves lymphocyte response against *H. pylori*

**Fig. 7:**
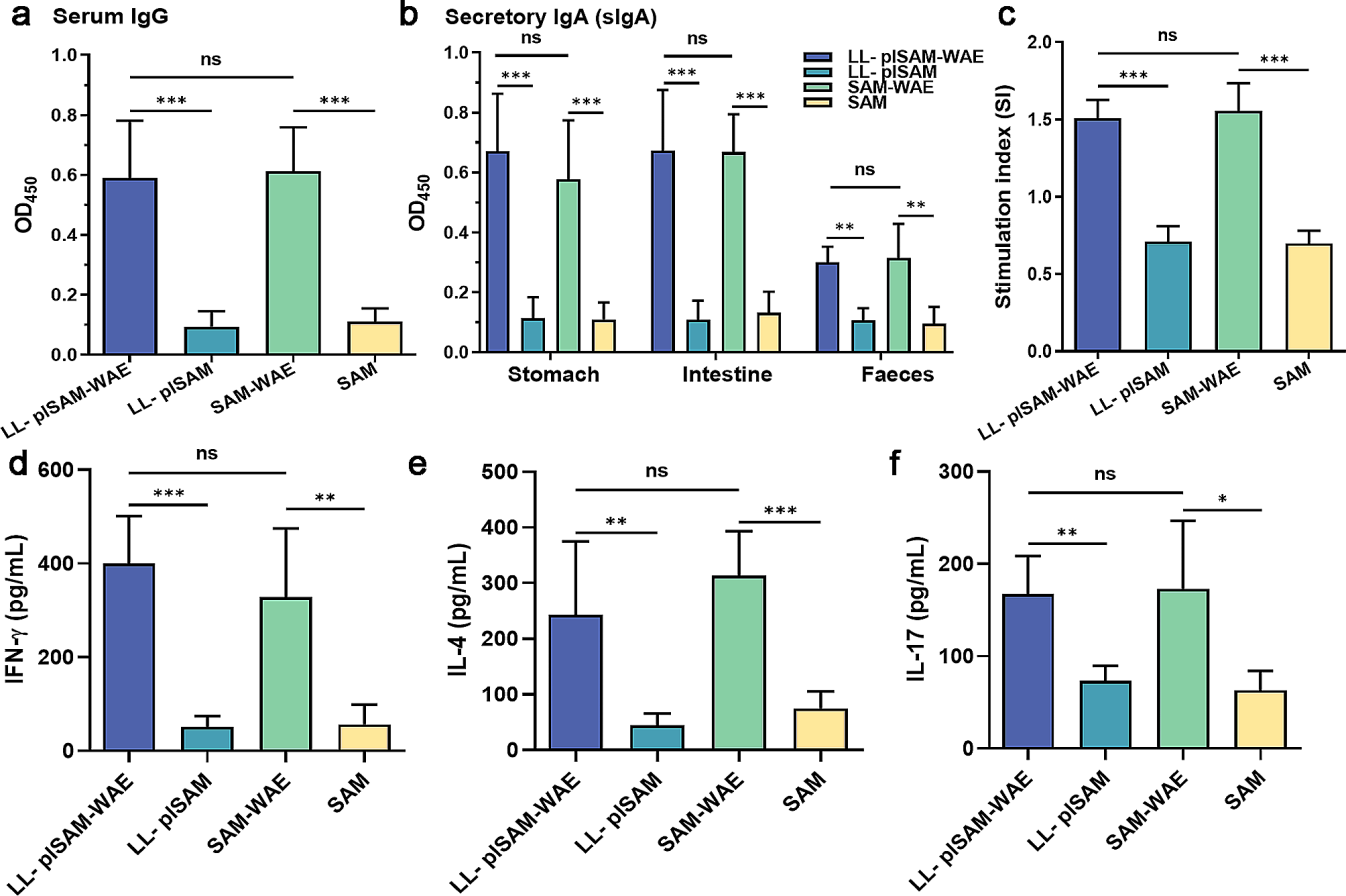
Detection of lymphocyte responses and antibodies specific for *H. pylori*. Samples of sera, stomach, intestine and feces were collected to identify the levels of serum IgG (**a**) and immunoglobulin A (**b**). Splenetic lymphocytes were isolated for proliferation analysis (**c**). ELISA was used to measure IFN-γ (**d**), IL-4 (**e**), and IL-17 (**f**) in the supernatant

We were curious whether LL-plSAM-WAE or SAM-WAE protein with PA may stimulate lymphocyte immunological responses. As a result, antibodies were detected in the serum, stomach, intestine, and feces of orally inoculated mice. Serum IgG and mucosal sIgA against *H. pylori* were clearly increased in those samples following oral stimulation with LL-plSAM-WAE or SAM-WAE protein, as shown in Fig. 7a and b. Furthermore, lymphocytes from LL-plSAM-WAE or SAM-WAE with PA-treated mice proliferated more than lymphocytes from LL-plSAM or SAM with PA- vaccinated animals (Fig. 7c). Furthermore, ELISA findings demonstrated that LL-plSAM-WAE or SAM-WAE with PA vaccination enhanced three cytokines (IFN-γ, IL-4, and IL-17) in splenic lymphocyte supernatant (Fig. 7d-f), which consistent with our previous research. Finally, the LL-plSAM-WAE or SAM-WAE protein increased the production of *H. pylori*-specific antibodies and promoted lymphocyte responses against *H. pylori* invasion.

## Discussion

*Helicobacter pylori* colonizes the human gastric mucosa and may lead to gastritis, ulcers, and even cancer [37]. Development of an effective *H. pylori* vaccine would be an important step in solving gastrointestinal diseases. We previously developed a multivalent epitope vaccination called CWAE that had multiple copies of certain B and Th cell epitopes, the cholera toxin B subunit (CTB), NAP, and other antigens [10]. Additionally, studies indicated that CWAE taken orally enhanced CD4^+^ T-cell responses and antibodies directed against *H. pylori* [38]. This research closely follows the our previously published work using a similar *L. lactis* surface expression system but with different combinations of *H. pylori* antigens (Urease, HpaA, HSP60, NAP) with a modified vector system in a mouse model.

The effectiveness of oral mucosal immunity is hampered by the poor bioavailability of protein antigens such as our polyvalent epitope vaccines due to the harsh environment of the gastrointestinal tract, pepsin decomposition, and mucosal clearance of foreign antigens, which induces immune tolerance rather than immune stimulation [39]. Effective vaccine delivery systems are required to overcome this natural barrier, and these should not only delivery a payload antigen, but also deliver to immune effector cells to activate the cellular and secretory IgA antibodies immune responses [40]. Designing targeting M cells that reside in the mucosa-associated lymphoid tissue is essential to improve the bioavailability of foreign antigens [41]. A small number of M cells scattered on the follicular-related epithelium take up antigen in the intestinal lumen through adsorption, pinocytosis, and transport them to the APCs in their pockets to initiate the intestinal mucosal immune response [42]. To create effective oral mucosa vaccines, several studies have examined immunological methods that target antigens to M cells. Antigen transport and the beginning of immune responses specific to an antigen are both influenced by the interaction between the M cell-targeting ligands Co1 and C5aR on M cells [43, 44], and the ligands Cpe and Claudin4 [28, 29, 45].

Based on the above foundation, researchers have concentrated on and achieved advancements in oral vaccination administration technologies [16]. *L. lactis* is a good example of an LAB that has been used to deliver oral vaccinations. *L. lactis* has thus far been utilized to express a number of foreign antigens, including bacterial antigens [46], virus antigens [47, 48], and parasite antigens [49]. Additionally, the display of vaccine antigens on the surface of *L. lactis* has drawn significant interest [50].

In this work, we created an M cell-targeting *L. lactis* surface display system for the gastrointestinal tract delivery of the vaccination antigen WAE. Additionally, WAE antigen was added to the system as LL-plSAM-WAE, and the NICE system was used to induce the production of SAM-WAE fusion proteins. According to the SDS-PAGE data, *L. lactis* was likely expressed recombinant proteins SAM-WAE after the addition of Nisin. Then, further ELISA evidence confirmed the SAM-WAE proteins expression on the surface of LL-plSAM-WAE. As for which expression mode of SAM-WAE is mainly in intracellular and bacterial surfaces, further related experiments should be explored. Additionally, the LL-plSAM-WAE reportedly elicited systemic, mucosal, and cell-mediated immune responses, there are two possibilities: on the one hand, although the recombinant proteins are rarely displayed on the surface of *lactic acid bacteria*, but it secreted enough to activate the immune response. On the other hand, the *L. lactis*, as a vaccine delivery system to elicit mucosal immune response, with the death of the LL-plSAM-WAE, which is targeted by M cells, followed by effective antigen presentation, thus triggering the immune response.

IgA-specific mucosal immune responses are effective against mucosal surface infections [51]. The molecular mechanism of *H. pylori* causing infection has not been fully clarified, and there is currently no vaccination that is especially effective against *H. pylori*. It is worth investigating novel methods of vaccination; oral administration is one of the constructive strategies as mentioned [52]. Prior research has shown the significance of antibody-mediated humoral immunity for defense against *H. pylori* infection [53]. However, further research revealed that an antibody-independent mechanism may also provide protection against *H. pylori* infection [54]. In our research, LL-plSAM-WAE or SAM-WAE protein administration in mice contributed to the generation of *H. pylori*-specific antibodies against urease, HpaA, HSP60 and NAP, and lymphocyte immune responses were promoted during *H. pylori* invasion. In addition, stomach, intestinal and faeces sIgA levels were elevated after oral immunization with LL-plSAM-WAE or SAM-WAE, compared to LL-plSAM or SAM. Based on these findings, we assume that LL-plSAM-WAE may play a protective role through humoral immunity and produce antibodies against *H. pylori*.

Given that M cells are specialized epithelial cells for the uptake of luminal antigens and possess a variety of APCs capable of transporting antigens to the underlying immune inductive organ of the mucosa and inducing antigen-specific immunity, a new delivery system containing M cell-targeting ligands would be valuable [55]. In contrast to CWAE, our investigation discovered that the SAM-conjugated multi epitope antigen CWAE, demonstrated good targeting capacity to M cells of ileum PPs. Additionally, the findings of the closed ileum loop experiment and immunohistochemistry analysis demonstrated that the peptide WAE present in the LL-plSAM-WAE and SAM-WAE proteins helped M cells and antigens co localize.

The role of the CD4^+^ T-cell (Th cell) response in defense against *H. pylori* infection has been previously described [56]. Host immunity and immunopathology events are fundamentally regulated by *H. pylori*-specific CD4^+^ T cells. It is known that Th1, Th2, Th9, Th17, Th22, and T regulatory (Treg) cells, either alone or in combination, may influence the outcome of a *H. pylori* infection [57, 58]. Previous studies have shown that, Treg and Th2 cells have anti-inflammatory effects when *H. pylori* infection occurs, however Th1 and Th17 cells may be either protective or harmful [59]. Th1 cells predominate in number among all stomach T cells obtained from *H. pylori*-infected individuals. More crucially, the antigen fragments included in the SAM-WAE vaccine (UreA_27–53_, UreB_158–251_ and UreB_321–385_) included a variety of known and projected CD4^+^ T-cell epitopes that might activate CD4^+^ T-cell responses specific to *H. pylori*. Interestingly, splenic lymphocytes isolated from LL-plSAM-WAE-immunized mice exhibited stronger proliferation ability after *H. pylori* infection, and the concentrations of IFN-γ, IL-17 and IL-4 increased to high levels. These findings showed that the immune defense mechanism of LL-plSAM-WAE may be producing specific sIgA and IgG antibodies against a number of *H. pylori* virulence proteins, as well as the immunological response of CD4^+^ T cells.

The ultimate goal of vaccines is to effectively prevent or treat microbial invasion and infection. Therefore, we constructed a therapeutic and preventive mouse model of *H. pylori* to evaluate the effect of the recombinant vaccine in the prevention of *H. pylori*, and the experimental results showed that oral with immunization LL-plSAM-WAE could significantly protect against *H. pylori* infection. The mechanism of prevention is mainly to activate the adaptive immune response, and produce serum IgG and mucosal IgA antibodies, and at the same time the body rapidly responds to inflammatory factors to defend against *H. pylori* infection, which plays a key role in the cellular immune response mediated by CD4 ^+ ^T cells.

Finally, at the population level, the application value of this *L. lactis* vaccine delivery system is mainly reflected in the following aspects. Firstly, compared to other host vectors, the use of *L. lactis* as mucosal vaccine vectors is a promising alternative, owing to their “generally regarded as safe” status, potential adjuvant properties, and tolerogenicity to the host [60]. Next, many investigations using *L. lactis* as cell factories for the production of specific antigen and testing the production of pharmaceutical products [61]. And the studies also found that the *L. lactis*-TB1-Co1 can induce elevations in mucosal as well as systemic immune reactions, and to a certain extent, provide protection against FMDV [48]. Therefore, using this vaccine carrier has a more long-term application prospect.

## Conclusion

In conclusion, we developed *L. lactis* LL-plSAM-WAE, a new vaccine delivery system with SAM-WAE antigen expressed on bacterial surfaces, which dramatically improved the ability to target M cells. Our research has shown that the M cell-targeting ligand SAM comprising Co1, Cpe, and CKS9 is a crucial development for *H. pylori* oral mucosal vaccinations. It is critical for the successful induction of mucosal and systemic immune responses in mice as well as the efficient transport of ligand-conjugated multi epitope antigens to mucosal immune components. Additionally, LL-plSAM-WAE oral vaccination increased the generation of antibodies against *H. pylori* virulence factors and the proliferation of T cells, providing immunological protection against *H. pylori* infection. An *L. lactis* based *H. pylori* vaccine is a strong candidate for progression to clinical trials.

### Electronic supplementary material

Below is the link to the electronic supplementary material.

Supplementary Material 1

## Data Availability

The data that support the findings of this study are in this published article and available from the corresponding author upon reasonable request.
