# Supplementary material for: Recombinant L. lactis vaccine LL-plSAM-WAE targeting four virulence factors provides mucosal immunity against H. pylori infection
Source: Microb Cell Fact. 2024 Feb 24;23:61. doi: 10.1186/s12934-024-02321-4 (PMC10893618; doi:10.1186/s12934-024-02321-4)
Supplement: Supplementary file 1 — Supplementary Material 1 [file 12934_2024_2321_MOESM1_ESM.rtf]

FASTA of the sequence in the manuscript
pNZ8148 plasmid (Accession: LC739717.1)
AGATCTAGTCTTATAACTATACTGACAATAGAAACATTAACAAATCTAAAACAGTCTTAATTCTATCTTGAGAAAGTATTGGTAATAATATTATTGTCGATAACGCGAGCATAATAAACGGCTCTGATTAAATTCTGAAGTTTGTTAGATACAATGATTTCGTTCGAAGGAACTACAAAATAAATTATAAGGAGGCACTCACCATGGAAAGAGGATCGCATCACCATCACCATCACGGATCTGGCTCTGGATCTGGTATCGAGGGAAGGCCTTATAATGGAACTGGATCCGACATTGTTATGACACAATCTCCGTCATTTCTATCTGCTTCAGTTGGAGATAGAGTCACAATTACTTGTAGAGCATCACAAGGCATTTCTAGTTACTTAGCTTGGTATCAACAGAAGCCTGGTAAAGCTCCAAAGTTACTTATCTATGCCGCAAGCACATTGCAATCAGGCGTTCCATCACGTTTCAGTGGGAGTGGATCAGGAACTGAATTTACACTAACAATAAGCTCATTACAACCAGAAGATTTTGCGACGTATTATTGCCAACAGTTGAATAGTTATCCTCCCAAATTTACCTTTGGTCCAGGAACAAAAGTCGAGATAAAACGAGAAGGGAAATCTAGTGGTTCAGGATCTGAGTCTAAAAGTGAAGTTCAGTTAGTTGAATCAGGTGGAGGATTGATTCAACCTGGTGGAAGTTTACGTCTTTCATGTGCAGCTTCAGGTCTTACCGTGAGTTCTAACTATATGTCATGGGTAAGACAAGCTCCTGGGAAAGGTTTAGAATGGGTTTCAGTTATCTATTCAGGTGGTTCTACATTCTATGCAGATAGCGTAAAAGGGCGATTTACGATTTCACGCGATAATTCAAAGAATACCTTGTACTTACAAATGAATAGCTTAAGAGCCGAAGATACTGCGGTATATTACTGTGCACGTGATCTTGACGTATATGGCTTAGATGTTTGGGGACAAGGAACTACTGTGACTGTGAGCTCTAAGCTTTCTTTGAACCAAAATTAGAAAACCAAGGCTTGAAACGTTCAATTGAAATGGCAATTAAACAAATTACAGCACGTGTTGCTTTGATTGATAGCCAAAAAGCAGCAGTTGATAAAGCAATTACTGATATTGCTGAAAAATTGTAATTTATAAATAAAAATCACCTTTTAGAGGTGGTTTTTTTATTTATAAATTATTCGTTTGATTTCGCTTTCGATAGAACAATCAAATCGTTTCTGAGACGTTTTAGCGTTTATTTCGTTTAGTTATCGGCATAATCGTTAAAACAGGCGTTATCGTAGCGTAAAAGCCCTTGAGCGTAGCGTGCTTTGCAGCGAAGATGTTGTCTGTTAGATTATGAAAGCCGATGACTGAATGAAATAATAAGCGCAGCGTCCTTCTATTTCGGTTGGAGGAGGCTCAAGGGAGTTTGAGGGAATGAAATTCCCTCATGGGTTTGATTTTAAAAATTGCTTGCAATTTTGCCGAGCGGTAGCGCTGGAAAATTTTTGAAAAAAATTTGGAATTTGGAAAAAAATGGGGGGAAAGGAAGCGAATTTTGCTTCCGTACTACGACCCCCCATTAAGTGCCGAGTGCCAATTTTTGTGCCAAAAACGCTCTATCCCAACTGGCTCAAGGGTTTGAGGGGTTTTTCAATCGCCAACGAATCGCCAACGTTTTCGCCAACGTTTTTTATAAATCTATATTTAAGTAGCTTTATTGTTGTTTTTATGATTACAAAGTGATACACTAATTTTATAAAATTATTTGATTGGAGTTTTTTAAATGGTGATTTCAGAATCGAAAAAAAGAGTTATGATTTCTCTGACAAAAGAGCAAGATAAAAAATTAACAGATATGGCGAAACAAAAAGGTTTTTCAAAATCTGCGGTTGCGGCGTTAGCTATAGAAGAATATGCAAGAAAGGAATCAGAACAAAAAAAATAAGCGAAAGCTCGCGTTTTTAGAAGGATACGAGTTTTCGCTACTTGTTTTTGATAAGGTAATATATCATGGCTATTAAAAATACTAAAGCTAGAAATTTTGGATTTTTATTATATCCTGACTCAATTCCTAATGATTGGAAAGAAAAATTAGAGAGTTTGGGCGTATCTATGGCTGTCAGTCCTTTACACGATATGGACGAAAAAAAAGATAAAGATACATGGAATAGTAGTGATGTTATACGAAATGGAAAGCACTATAAAAAACCACACTATCACGTTATATATATTGCACGAAATCCTGTAACAATAGAAAGCGTTAGGAACAAGATTAAGCGAAAATTGGGGAATAGTTCAGTTGCTCATGTTGAGATACTTGATTATATCAAAGGTTCATATGAATATTTGACTCATGAATCAAAGGACGCTATTGCTAAGAATAAACATATATACGACAAAAAAGATATTTTGAACATTAATGATTTTGATATTGACCGCTATATAACACTTGATGAAAGCCAAAAAAGAGAATTGAAGAATTTACTTTTAGATATAGTGGATGACTATAATTTGGTAAATACAAAAGATTTAATGGCTTTTATTCGCCTTAGGGGAGCGGAGTTTGGAATTTTAAATACGAATGATGTAAAAGATATTGTTTCAACAAACTCTAGCGCCTTTAGATTATGGTTTGAGGGCAATTATCAGTGTGGATATAGAGCAAGTTATGCAAAGGTTCTTGATGCTGAAACGGGGGAAATAAAATGACAAACAAAGAAAAAGAGTTATTTGCTGAAAATGAGGAATTAAAAAAAGAAATTAAGGACTTAAAAGAGCGTATTGAAAGATACAGAGAAATGGAAGTTGAATTAAGTACAACAATAGATTTATTGAGAGGAGGGATTATTGAATAAATAAAAGCCCCCCTGACGAAAGTCGACGGCAATAGTTACCCTTATTATCAAGATAAGAAAGAAAAGGATTTTTCGCTACGCTCAAATCCTTTAAAAAAACACAAAAGACCACATTTTTTAATGTGGTCTTTATTCTTCAACTAAAGCACCCATTAGTTCAACAAACGAAAATTGGATAAAGTGGGATATTTTTAAAATATATATTTATGTTACAGTAATATTGACTTTTAAAAAAGGATTGATTCTAATGAAGAAAGCAGACAAGTAAGCCTCCTAAATTCACTTTAGATAAAAA

 
SAM(887 bps)£ºCATATGACTACTTATACCGTCAAATCTGGTGATACTCTTTGGGGAATCTCACAAAGATATGGAATTAGTGTCGCTCAAATTCAAAGTGCGAATAATCTTAAAAGTACCATTATCTACATTGGTCAAAAACTTGTACTGACAGGTTCAGCTTCTTCTACAAATTCAGGTGGTTCAAACAATTCCGCAAGCACTACTCCAACCACTTCTGTGACACCTGCAAAACCAACTTCACAAACAACTGTTAAGGTTAAATCCGGAGATACCCTTTGGGCGCTATCAGTAAAATATAAAACTAGTATTGCTCAATTGAAAAGTTGGAATCATTTAAGTTCAGATACCATTTATATTGGTCAAAATCTTATTGTTTCACAATCTGCTGCTGCTTCAAATCCTTCGACAGGTTCAGGCTCAACTGCTACCAATAACTCAAACTCGACTTCTTCTAACTCAAATGCCTCAATTCATAAGGTCGTTAAAGGAGATACTCTCTGGGGACTTTCGCAAAAATCTGGCAGCCCAATTGCTTCAATCAAGGCTTGGAATCATTTATCTAGCGATACTATTTTAATTGGTCAGTATCTACGAATAAAAGGTACCACTAGTTCTAGATGTAAATCAACACATCCTTTATCATGTTCATTTCATCAATTACCTGCAAGAAGTCCTTTACCATCATTAGATGCAGGACAATATGTTTTAGTTATGAAAGCAAATTCAAGTTATTCAGGTAATTATCCATATTCAATTTTATTTCAAAAATTTTGAAAGCTT

WAE (1614 bps):
GGTACCATGAAGACCTTTGAAATCCTGAAACACCTGCAAGCCGACGCCATTGTCCTGTTTATGAAAGTCCACAACTTCCATTGGAACGTGAAAGGTACGGACTTTTTCAACGTGCATAAAGCCACCGAAGAAATCTACGAAGAATTCGCTGATATGTTCGATGACCTGGCGGAACGCATTGTTCAGCTGGGCCATCACCCGCTGGTCACGCTGAGCGAAGCGATCAAACTGACCCGTGTTAAAGAAGAAACCAAGACGAGCTTTCACTCTAAAGACATCTTCAAGGAAATCCTGGAAGATTACAAGTACCTGGAAAAGGAATTCAAGGAACTGAGCAATACCGCCGAAAAAGAAGGTGATAAGGTGACCGTTACGTATGCTGATGACCAGCTGGCGAAACTGCAAAAGTCTATTTGGATGCTGCAGGCACATCTGGCTGATCCGCGCGTGCCGAGCTCTGGTATCAAACTGAACTATGTTGAAGCGGTCGCCCTGATTAGTGCCCACATCATGGAAGAAGCACGTGCTGGTAAAAAGACCGGCATTAAACTGAATTACGTGGAAGCGGTTGCCCTGATTTCAGCGCATATCATGGAAGAAGCACGCGCTGGTAAAAAGACCTCGGTTGAACTGATTGATATCGGCGGTAACCGTCGCATTTTTGGTTTCAATGCTCTGGTCGACGGCAGTTCCGTGGAACTGATCGACATCGGCGGTAATCGTCGCATCTTTGGTTTCAATGCACTGGTCGATGGCTCTGACCCGAAACGTACGATTCAGAAAAAGAGCGGTTCTGACCCGAAGCGCACCATCCAGAAAAAATCTGGCTCCGATCCGAAACGTACCATTCAGAAAAAGTCAGGTTCGGTGGAAGGCATGCAATTTGATCGCGGTTATCTGTCACCGTACTTTGGTTCGGTTGAAGGCATGCAGTTCGACCGTGGCTATCTGAGTCCGTACTTTGGCGGTGGCACCGGTCCGGCAGATGGCACGAACGCAACCACGATTACCCCGGGTCGTCGCAATCTGAAATGGATGCTGCGTGCGGCCGAAGAATATTCCATGAACCTGGGTTTTCTGGCAAAAGGCAACGCTAGTAATGACGCGTCCCTGGCCGATCAGATTGAAGCGGGTGCCATTGGCTTCAAAATCCATGAAGATTGGGGCACCACGCCGAGCGCGATCAATCACGCCCTGGATGTCGCAGACAAATACGATGTCCAAGTGGCCATCCATACCGACACGGGCAGTTGCCATCACCTGGATAAATCCATTAAGGAAGACGTGCAGTTTGCAGATTCACGTATTCGCCCGCAAACGATCGCAGCTGAAGACACCCTGCACGATATGGGCATTTTCAGCATCACCTCATCGGATTCTCAGGCGATGGGTCGTGTCGGCGAAGTCATTACGCGCACCTGGCAGACGGCGGATAAAAACAAGAAGTCTAGATGTAAATCAACACATCCTTTATCATGTTCATTTCATCAATTACCTGCAAGAAGTCCTTTACCATCATTAGATGCAGGACAATATGTTTTAGTTATGAAAGCAAATTCAAGTTATTCAGGTAATTATCCATATTCAATTTTATTTCAAAAATTTTGAAAGCTT


Figure S1. Detection of T-cell immune responses in the MLNs. (a) Shown were representative FACS plots in MLNs. (b) The single cell suspensions from the MLN that were activated in vitro with 10 μg/mL antigen are shown in representative ELISPOT data. (c) The quantity of splenic IFN-γ producing cells that are antigen-specific.


Figure S2 Antiserum vaccinated with NZ9000 specificity to antigens for negative control. Western blot results showed that sera of mice vaccinated with Lactobacillus NZ9000 failed to specifically recognize antigens Urease, UreA, UreB, HpaA and NAP.
